# Anthropometric Measurements: Options for Identifying Low Birth Weight Newborns in Kumasi, Ghana

**DOI:** 10.1371/journal.pone.0106712

**Published:** 2014-09-16

**Authors:** Easmon Otupiri, Priscilla Wobil, Samuel Blay Nguah, Michelle J. Hindin

**Affiliations:** 1 Kwame Nkrumah University of Science and Technology, Kumasi, Ghana; 2 Komfo Anokye Teaching Hospital, Kumasi, Ghana; 3 Johns Hopkins Bloomberg School of Public Health, Baltimore, Maryland, United States of America; National Taiwan University Hospital, Taiwan

## Abstract

**Background:**

In Ghana, 32% of deliveries take place outside a health facility, and birth weight is not measured. Low birth weight (LBW) newborns who are at increased risk of death and disability, are not identified; 13%–14% of newborns in Ghana are LBW. We aimed at determining whether alternative anthropometrics could be used to identify LBW newborns when weighing scales are not available to measure birth weight.

**Methods:**

We studied 973 mother and newborn pairs at the Komfo Anokye Teaching and the Suntreso Government hospitals between November 2011 and October 2012. We used standard techniques to record anthropometric measurements of newborns within 24 hours of birth; low birth weight was defined as birth weight <2.5kg. Pearson's correlation coefficient and the area under the curve were used to determine the best predictors of low birth weight. The sensitivity, specificity and predictive values were reported with 95% confidence intervals at generated cut-off values.

**Results:**

One-fifth (21.7%) of newborns weighed less than 2.5 kg. Among LBW newborns, the following measurements had the highest correlations with birth weight: chest circumference (r = 0.69), mid-upper arm circumference (r = 0.68) and calf circumference (r = 0.66); the areas under the curves of these three measurements demonstrated the highest accuracy in determining LBW newborns. Chest, mid-upper arm and calf circumferences at cut-off values of ≤29.8 cm, ≤9.4 cm and ≤9.5 cm respectively, had the best combination of maximum sensitivity, specificity and predictive values for identifying newborns with LBW.

**Conclusions:**

Anthropometric measurements, such as the chest circumference, mid-upper arm circumference and calf circumference, offer an opportunity for the identification of and subsequent support for LBW newborns in settings in Ghana, where birth weights are not measured by standardized weighing scales.

## Introduction

The Millennium Development Goal 4 - to reduce deaths of children under-five years by two thirds - may be unattainable without halving newborn deaths, which now comprise 40% of all under-five deaths globally [1]–[3]. In order to meet the MDG-4, Ghana would have to reduce the current under-five mortality rate to 40/1000 live births by 2015 [4].

A major risk factor for neonatal mortality is low birth weight (LBW); a birth weight less than 2500 g. Every ten seconds, an infant from a developing country dies from a disease or infection that can be attributed to LBW [5]. Nearly all of the newborns who die are LBW, and are mostly in rural communities [6]. More than half of these LBW babies die shortly after birth at home [3], [7], [8], mostly in rural families [9].

A World Health Statistics report asserted that 15% of babies are born worldwide with LBW [10]. In sub-Saharan Africa, very similar figures were also reported with a LBW rate of 14%, and for Ghana 13% [10]–[12]. These figures are mainly facility-based and could under-estimate the burden of LBW. Despite the fact that available reports suggest LBW babies constitute only about 13%–15% of children born, they account for 60%–90% of neonatal deaths [6], [13]. The 2008 Ghana Demographic and Health Survey reports that among babies assessed by mothers as ‘small or very small,’ infant mortality and neonatal mortality were twice as high when compared with babies assessed to be ‘average or large’ at birth. Only 43% of newborns have their birth weights recorded, and 14% of all live births are small; 15% (rural) versus 11% (urban) [14].

In Ghana, 32% of deliveries occur outside a health facility [15], and the gestational ages of these newborns are also not known or documented [13], [16], and are therefore not identified for supportive interventions such as Kangaroo Mother Care, extra support in feeding, infection prevention and sepsis management.

The goal of this study was to evaluate simple anthropometric measurements against the “gold standard” of assessing low birth weight using a calibrated scale in order to provide simple measurements that can be used to guide community health workers and unskilled delivery attendants to identify low birth weight babies, and promote essential newborn care.

### Study sites

The Komfo Anokye Teaching Hospital (KATH), a tertiary health institution in Kumasi located in the Subin sub-metropolis was one of the two study sites. The Suntreso Government Hospital, a primary level referral facility located in the Bantama sub-metropolis of Kumasi was the second site.

KATH operates as a national referral centre for the northern sector of Ghana. The Child Health Department of KATH has a Paediatric Emergency Unit, three specialist wards and a Mother Baby Unit (MBU). The MBU admits infants less than two months old. This unit has three main wings – a High Dependency Unit (HDU), a LBW and Kangaroo Mother Care Unit (KMC) and a Septic Unit. The HDU admits newborns who need special care and are referred from labor wards within KATH and surrounding district and private hospitals. The LBW and KMC units admit preterm and low birth weight babies for special care and KMC. The Septic Unit admits babies up to two months of age referred from the outpatient department of KATH and other hospitals with suspected sepsis. Mothers are accommodated in a separate room, but on the same floor of the unit. From the MBU records, the average monthly admission is about 150 cases, of which 90 are neonates (0–28 days). The 2010 MBU admission and discharge records show that low birth weight accounts for about 25% of admissions. According to the KATH labor ward records for 2010, there were about 13,000 deliveries, and approximately 1000 were low birth weight, accounting for 9% of all deliveries.

The Suntreso Government Hospital (SGH) is a district hospital within the Bantama sub-metropolis, with 238 health facilities within its catchment area. These include private hospitals, maternity homes, pharmacy shops and community-based health planning and services (CHPS) zones. The hospital runs a 24-hour service with several units. The Maternity Unit of this hospital has an antenatal clinic, a labor ward, a theatre and a postnatal ward. There are 28 midwives in the unit and it records about 2,500 deliveries per year. About 5% of all live births are low birth weight. The MBU admits sick newborns delivered at SGH. Others are referred from the health facilities within the Bantama sub-metropolis. In order to reduce the congestion at the KATH MBU, moderately sick newborns are transferred to the SGH MBU to continue with their treatment; low birth weight accounts for 15% of admissions.

## Study Methods

### Ethics statement

The study was undertaken according to the tenets of internationally accepted Good Clinical Practice (GCP). The Committee on Human Research, Publications and Ethics (CHRPE) of the Kwame Nkrumah University of Science and Technology, and the Komfo Anokye Teaching Hospital approved the study (CHRPE/AP/155/12). A participant information leaflet/consent form was read aloud to the potential participant. If the mother/guardian did not understand English, it was read in the local language of her choice in the presence of a witness. The parent or guardian indicated their willingness to participate in the study with their newborn by signing and dating or thumb-printing the consent form. It was explained to parents/guardians that there would be some discomfort to the baby during the anthropometric measurements, and that trained health workers would undertake the measurements in order to minimize discomfort. It was also explained to parents/guardians that participating in the study was voluntary, and they had the right to discontinue at any time without any consequences to their newborn or themselves.

### Study design

We conducted an observational study that used a cross-sectional analytical design to study newborns delivered at or admitted to two public hospitals - Komfo Anokye Teaching Hospital and Suntreso Government Hospital in Kumasi, Ghana between November 2011 and October 2012.

### Study population

All mothers and their newborns delivered at or admitted to KATH and SGH.

### Inclusion criteria

Newborns aged less than 24 hours, and who did not have major congenital malformations and/or birth injuries such as scalp swellings and limb fractures, and who were not less than 1.0 kg and very sick needing oxygen therapy.

### Sample size

The sample size was determined by making assumptions mostly based on the area under the curve and correlation findings by Sreeramareddy and others [17]. Based on load at the MBU and postnatal wards of the two study hospitals, we assumed that 10%–20% of the newborns to be recruited would be low birth weight. Using a correlation of 0.75 between two continuous anthropometric measurements, a power of 80%, a type I error of 0.05, and a two sided z-test, we determined that 179 LBW babies and 716 normal birth weight babies were required [18] to detect a difference of 0.05 in the area under the curve (AUC) between two continuous anthropometric tests with AUCs of 0.80 and 0.85. To account for an expected 5% incomplete data, at least 940 neonates were required for the study. At the end of the study, a total of 973 newborns had been recruited into the study.

### Recruitment

Based on the KATH and SGH records, about 15,000 children are born annually at these hospitals. This translates into about 1,200 deliveries each month. Since the study had a 12-month duration and the representative sample was 940 babies, we considered recruiting at least 78 newborns each month into the study. In order to meet the study duration and also to remove the effect of season of delivery, newborns were recruited throughout the 12-month study period. The assumption was that in each month, out of the approximately 1,200 deliveries, one-in-two mothers would meet the study criteria, would not be discharged home before recruitment, and would also consent to participate, so we expected about 600 mothers each month; we used systematic random sampling method for selection of mother and newborn pairs. Every seventh mother who delivered at KATH or SGH during any given month was approached for recruitment into the study. When the monthly sample of 78 was obtained, recruitment for that month ceased. If for any reason, the monthly sample for any given month was not obtained, the sampling interval for the ensuing month was reduced and when the expected enrolment was achieved, the subsequent month returned to the initial sampling interval.

### Study procedure

Four research assistants who were health care assistants and nurses on the two MBUs and postnatal wards were trained to measure birth weight, length, head circumference, chest circumference, mid-upper arm circumference, thigh circumference, calf circumference and foot length. Two pediatricians supervised the research.

Study participants were recruited from the postnatal wards and the MBUs of the two public hospitals. Midwives assessed the babies on the labor ward and if they were stable and well, they were transferred with their mothers to the postnatal ward and discharged in six hours. However, if a baby was assessed and found to be sick or at high risk of becoming sick, the baby was referred to the MBU for evaluation and management. This was part of the routine care provided for all mothers and babies.

The research assistants screened the babies on the MBU and postnatal wards on a daily basis for inclusion into the study; we screened 1,090 newborns within 24 hours of birth.

### Data collection

Information on mother's demographics, pregnancy and delivery history were obtained verbally from the mother or guardian, and any additional information were retrieved from the antenatal card and delivery records. Specific information on the mother's demographic characteristics obtained included: age, telephone number, place of residence, home address (with land marks), educational status, occupation, ethnicity, religion, marital status, and household income.

Variables on mother's pregnancy and delivery history included gravidity, parity, last menstrual period (LMP), expected date of delivery, mode of delivery, date and time of delivery, baby's weight, sex, Apgar scores and gestational age. The gestational age was used to determine whether the newborn was term or preterm and was assessed by the mother's LMP and/or ultrasound report.

The newborn's anthropometric measurements were taken within the first 24 hours of life. The methods and techniques used were based on standardized techniques and criteria described in published studies [19]–[23]. We measured the birth weights of all newborns to the nearest 100 g using Model 180 Salter weighing scale (England), calibrated with a bottle weighing 1000 grams as was done routinely for all babies delivered at the two hospitals. The body length and the foot length (FL), as well as circumferences of head (OFC), chest (ChC), thigh (ThC) and calf (CaC) were measured to the nearest 0.1 cm using a non-elastic, flexible measuring tape, while the MUAC was measured using the UNICEF tri-coloured flexible measuring tape. For each newborn, the length, OFC, ChC, MUAC, ThC, CaC and FL were measured three times by the research assistant. While one research assistant was taking the measurements, one research assistant was recording the measurements. The mean of each of the sets of measurements was used for analysis.

### Data analysis

The data were entered into a database in EpiData version 3.1 and then transferred to STATA IC version 11.1 (StataCorp Lp Texas, 2010) for analyses. Frequencies for the demographic characteristics, pregnancy and delivery history were determined. Continuous variables such as the birth weight and anthropometric measurements were reported using mean and standard deviation if not skewed. All anthropometric measurements were measured three times and their means were used in further analysis after being cleaned of abnormal values. Between-gender comparisons of continuous variables were performed using independent sample t-test. Pearson's correlation coefficient was determined between the various anthropometric measurements.

Multiple methods were used to assess the accuracy of the anthropometric measures against weight taken using a calibrated scale for identifying the baby as “low birth weight”. First, pairwise correlation between birth weight groups and anthropometric measurements was determined. Second, non-parametric receiver operating characteristic (ROC) curve analysis was done, and ROC curves were used to evaluate the accuracy of various anthropometric measurements to predict LBW, indicated by the area under the curve (AUC). Next, pairwise comparison of the AUC of the measurements was done to determine the one with the highest AUC. Finally, sensitivity, specificity, negative and positive predictive values were calculated at all cut-off points for any anthropometric measurement. We chose as “optimum,” the cut-off points with the highest [(sensitivity + specificity)/2] ratio, at which there was a maximal correct classification of the anthropometric measurement. The sensitivity, specificity and predictive values were reported with 95%CI using these generated cut-offs.

## Results

### Study flowchart

**Figure 1:**
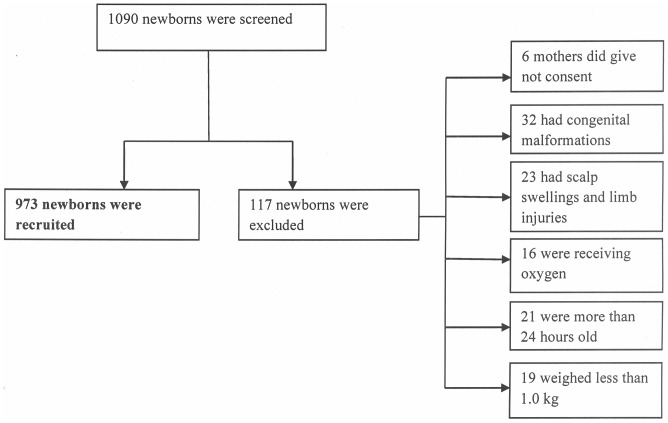
Study flowchart.

A total of one thousand and ninety (1090) newborns were screened, 117 (11.7%) were excluded while 973 (89.3%) were recruited between November 2011 and October 2012. Of the 117 who were excluded, 5% of mothers did not give consent, 27.4% had congenital malformations, 19.7% had scalp swellings and/or limb injuries, 13.7% were critically ill and receiving oxygen, 17.9% were more than 24 hours old and 16.2% weighed less than 1.0 kg. [Fig. 1]

### Sample characteristics

In terms of numbers, there was nearly the same number of male babies as there was for female babies in the study sample. A total of 211 (21.7%) newborns weighed less than 2.5 kg (LBW) and 762 (78.2%) were normal birth weight (NBW). Out of the 211 LBW (<2.5 kg) newborns, 183 (18.8%) weighed between 1.5 kg and 2.4 kg, while 28 (2.9%) were very low birth weight (VLBW) and weighed less than 1.5 kg. The overall mean birth weight was 2.9 kg with a standard deviation (SD) of 0.7. The mean weight of males (2.9 kg, SD 0.7) was significantly higher than that of females (2.8 kg, SD 0.7). Regarding gestational age of the newborns, 201 (20.7%) were preterm.

Mothers had a modal age of 25–29 years, and 15.6% of mothers did not have any formal education at all. Most women (45.5%) were into sales and services or trading, while 9.9% were unemployed. Over 80% of mothers were either married (60.5%) or living together with their partner (25.1%). [Table 1]

**Table 1:** Maternal characteristics.

Characteristic	Number (%)
Age group (years):	
<20	64 (6.6)
20–24	202 (20.8)
25–29	336 (34.5)
30–35	260 (26.7)
<35	111 (11.4)
Education level:	
None	152 (15.6)
Primary	181 (18.6)
Middle/JHS	414 (42.6)
Secondary/SHS	117 (12.0)
Higher than secondary	109 (11.2)
Occupation:	
Professional/Technical/Managerial	128 (13.2)
Clerical/Secretarial	16 (1.6)
Sales & services/Trader	443 (45.5)
Skilled manual	207 (21.3)
Unskilled manual	52 (5.3)
Agriculture/farming	31 (3.2)
Unemployed	96 (9.9)
Marital status:	
Never married	135 (13.9)
Married	589 (60.5)
Living together	244 (25.1)
Divorced/Separated	4 (0.4)
Widowed	1 (0.1)

### Pairwise correlation between birth weight and anthropometrics

Birth weight correlated significantly with all the anthropometric measurements. Among LBW newborns, the highest correlations with birth weight were: ChC (r = 0.69), MUAC (r = 0.68), CaC (r = 0.66), and ThC (r = 0.65). [Table 2]

**Table 2:** Pairwise correlation between birth weight groups and various anthropometric measurements.

	r (p-value)		
Measurement (cm)	NBW≥2.5 kg	LBW<2.5 kg	VLBW<1.5 kg
**ChC**	0.49 (0.00)	**0.69 (0.00)**	0.30 (0.12)
**MUAC**	0.45 (0.00)	**0.68 (0.00)**	0.21 (0.29)
**CaC**	0.50 (0.00)	**0.66 (0.00)**	0.15 (0.44)
**ThC**	0.42 (0.00)	**0.65 (0.00)**	0.28 (0.16)
OFC	0.38 (0.00)	0.58 (0.00)	0.33 (0.83)
FL	0.23 (0.00)	0.53 (0.00)	0.44 (0.02)
Length	0.30 (0.00)	0.52 (0.00)	0.24 (0.22)

### Accuracy of anthropometrics

To assess how accurately the anthropometrics matched up to the gold standard (low birth weight measured using a weighing scale), AUC analysis was conducted. Using chest circumference (ChC) to identify LBW newborns proved to be the most accurate based on the fact that it had the highest AUC of 0.91 (95% CI: 0.89–0.94). Mid upper arm circumference (AUC = 0.90, 95% CI 0.88–0.93) and calf circumference (AUC = 0.90, 95% CI 0.87–0.92) were also very accurate. Other measures were less accurate, with the least accurate being foot length (FL) (AUC = 0.74, 95% CI 0.70–0.78). [Table 3]

**Table 3:** Distribution of the area under the curve (AUC) for various anthropometric measurements.

Measurements (cm)	LBW (AUC, 95% CI)
**Chest Circumference (ChC)**	**0.91 (0.89–0.92)**
**Mid-Upper Arm Circumference (MUAC)**	**0.90 (0.88–0.93)**
**Calf Circumference (CaC)**	**0.90 (0.87–0.92)**
Thigh Circumference (ThC)	0.87 (0.84–0.90)
Head Circumference (OFC)	0.85 (0.82–0.88)
Length	0.82 (0.79–0.85)
FL	0.74 (0.70–0.78)

In order to assess whether the most accurate measure, chest circumference, compares with the other anthropometrics, the AUC of chest circumference was compared with the AUC estimates of all anthropometric measurements in this study using Pearson's pairwise correlation method. When the p-values are not statically significant (p<0.05), the interpretation is that the AUC is no different from the chest circumference. As shown, the AUCs of the MUAC (p = 0.51) and of CaC (p = 0.20) were not significantly different from ChC in identifying LBW newborns. After adjusting for multiple comparisons, there were still no significant differences between the AUC of ChC and that of MUAC (p = 1.00) and CaC (p = 1.00). However, there were significant differences between the AUC of ChC and the AUC of ThC, OFC, length and FL. [Table 4]

**Table 4:** Comparison of the AUC of chest circumference and AUC estimates of other anthropometric measurements among LBW babies.

	AUC	SE	p-value	Bonferroni Corrected p-values*
**ChC**	**0.91**	0.01	**-**	**-**
**MUAC**	**0.90**	0.01	**0.51**	**1.00**
**CaC**	**0.90**	0.01	**0.20**	**1.00**
ThC	0.87	0.01	0.00	0.03
OFC	0.85	0.02	0.00	0.00
Length	0.82	0.02	0.00	0.00
FL	0.74	0.02	0.00	0.00

* ***p-value adjusted for multiple comparisons using Bonferroni's Method.***

### Predictive values of anthropometric measurements that identify LBW newborns

A ChC at a cut-off value of ≤29.8 cm had the highest positive predictive value (PPV) of 75.0% (95%CI:68.5%–80.7%) for identifying LBW newborns. Although a thigh circumference (ThC) with a cut-off value of ≤13.3 cm had the highest negative predictive value (NPV) of 93.2% (95%CI:91.0%–95.0%), all the other measurements also had high NPVs of 85% or above. [Table 5]

**Table 5:** Predictive values of anthropometric measurements that identify LBW newborns.

Anthropometric measurements	Cut-off value (cm)	PPV % (95% CI)	NPV % (95% CI)
**ChC**	≤29.8	**75.0 (68.5–80.7)**	**92.8 (90.7–94.5)**
**CaC**	≤9.5	**72.7 (66.2–78.6)**	**92.3 (90.2–94.1)**
**MUAC**	≤9.4	**72.7 (66.0–78.7)**	**91.9 (89.8–93.8)**
OFC	≤31.0	66.3 (59.4–72.8)	90.0 (87.7–92.0)
FL	≤7.4	63.0 (54.2–71.1)	85.0 (82.4–87.3)
ThC	≤13.3	51.5 (46.0–57.1)	93.2 (91.0–95.0)
Length	≤46.0	42.2 (37.3–47.3)	92.1 (89.6–94.2)

## Discussion

In this study, ChC, MUAC and CaC were equally and highly accurate in identifying LBW newborns. A meta-analysis by Goto [24], studies by Ghosh & others [25] in a tertiary hospital in Kolkata, and Sreeramareddy & others [17] in a regional hospital in Nepal, all confirm these findings, except that CaC was not investigated in those studies.

The measurement of ChC at the nipple line makes the method simple, reliable and operationally feasible. On the other hand, it requires the removal of the newborn's clothes in order to expose the trunk, increasing the risk of hypothermia. Per contra, measurements of MUAC and CaC do not require much exposure of the newborn, and therefore the risk of hypothermia is reduced. Maintaining the “warm chain,” and taking the measurement quickly could address these concerns with the measurement of ChC. Since the accuracy of ChC, MUAC and CaC were not significantly different in determining LBW, MUAC and CaC could be used if there are serious concerns with measuring ChC in the community setting. The measuring tapes used for these measurements are inexpensive, easy to carry around and maintain, and can be easily used by community health workers [17], [23], [26]–[29]. The appropriate method of measuring anthropometrics needs further confirmation in a community setting.

We suggest that cutoffs could be provided for community health workers and others attending deliveries to suggest which babies are most at risk. Based on the data from this study, a chest circumference ≤30, calf and mid upper arm circumferences ≤9.5 are concerning. Before providing national recommendations, further validation is required.

One pertinent question that needs to be answered is, do we use all the best correlates and highly accurate measurements (ChC, MUAC and CaC) observed in this study to identify LBW or chose a single measurement? Some studies [25], [29] have used a combination of measurements and developed a normogram or formula that was used to identify LBW babies with good validity and reliability. Others have also used a single method [22], [28], with equally good results. It implies that, whatever method is chosen to be used in the community setting would have to be tested, and should be operationally feasible and reliable within the chosen setting.

### Correlation between birth weight and anthropometrics

Many studies have reported strong positive correlations between birth weight and several anthropometrics: 0.60–0.97 [19], [24], [26], [29]. Some studies found the correlation to be highest with MUAC, with estimates ranging from r = 0.66 to 0.95 and ChC (r = 0.60–0.85) [19], [25], [29], while others reported that ChC, OFC and ThC had the highest correlations [27], [28], and Das & others added CaC (r = 0.95) [30]. These findings are similar to those of our study – ChC (r = 0.690, MUAC (r = 0.68), CaC (r = 0.66) and ThC (r = 0.65). Not only did these measurements correlate strongly and positively with birth weight, they also correlated positively with each other as well. FL showed the weakest correlation with birth weight in our study and in other studies [19].

### Sensitivity and specificity of anthropometric measurements that identify LBW babies

Any anthropometric measurement that is chosen as an alternative to birth weight should be very sensitive so that majority of LBW newborns would be identified for referral and extra care. At the same time it has to be highly specific in order to prevent unnecessary referrals of normal weight babies to higher level centres.

Most studies have used various cut-off points ranging from 29.5 cm to 33.5 cm for ChC for predicting LBW newborns [17], [19], [21], [25]. Results from this study showed that a ChC of ≤29.8 cm had a sensitivity of about 74% and a specificity of about 93%. This means that, seven out of ten newborns who are LBW would be correctly identified using ChC, and only three out of the ten who were normal weight would be wrongly classified. A study by Rustagi & others [19] reported similar sensitivity values of 75%, while other studies reported higher sensitivities between 88% and 97% [17], [21], [25].

Several other studies selected cut-off values for MUAC ranging between 9.0 cm and 10.0 cm, which were comparable to the MUAC cut-off point of 9.4 cm observed in our study [19], [24], [26], [28], [29]. According to a meta-analysis by Goto, very few studies investigated CaC [26]. One study reports a CaC with a cut-off point of 9.7 cm; this similar to the cut-off value of 9.5 cm observed in our study [31].

Results from our study further showed that MUAC and CaC had sensitivities of 71% and 72% respectively, with each having a specificity of approximately 93%. These figures are very similar to that for ChC in our study. Comparatively, some studies [19], [31] have reported similar sensitivities of 74% and 75% while others [25] have observed higher sensitivities but with low predictive values, and hence high false positive rates.

### Sample characteristics

In this study, the number of baby boys and the number of baby girls was almost the same, and this finding is very similar to several studies conducted in Africa and Asia [17], [22], [27], [28], [31]–[33]. Comparable to studies in Nigeria [32] and Asia [22] one-in-five newborns was LBW. The prevalence of LBW in our study was higher than what was observed in other studies that reported a prevalence of 3.8%–18% [17], [20], [22], [27], [28], [32], [33]. On the other hand, the prevalence of LBW observed in this study was also found to be relatively lower than what was reported by others: 28%–49% [21], [30], [32]. The small proportion of preterm to term babies in these studies accounts for the differences in LBW prevalence. In certain studies where they included only term babies [17], [27] or where few were preterm [33], the mean birth weight of newborns in those studies was relatively higher (3.0 kg±0.4 to 3.1 kg±0.5) than in our study (2.9 kg±0.7) where one-in-five newborns was preterm.

## Conclusions

To conclude, ChC, MUAC and CaC measurements may offer a great opportunity for the identification of, and subsequent support for low birth weight newborns in areas in Ghana where birth weights are not measured. The Ghana Health Service may consider supporting community-based studies to test the validity and reliability of these anthropometric measurements for use in community settings in Ghana, where birth weight is not likely to be checked. Anthropometric measurements will contribute to obtaining a more comprehensive measurement, and account of the burden of low birth weight especially in the hard-to-reach areas and in marginalized populations.

### Limitations of the study

Our findings and recommendations should be read against the backdrop of certain potential limitations. The study was hospital-based and therefore may not be representative of the children born outside a health facility. Comparing studies with predictive values presents its own challenges as predictive values depend on prevalence, which could vary between studies without affecting the sensitivity/specificity of the measurements.
